# Spatial utilization predicts animal social contact networks are not scale-free

**DOI:** 10.1098/rsos.171209

**Published:** 2017-12-13

**Authors:** Alex James, Jeanette C. McLeod, Carlos Rouco, Kyle S. Richardson, Daniel M. Tompkins

**Affiliations:** 1 Biomathematics Research Centre, University of Canterbury, Christchurch, New Zealand; 2 Te Pūnaha Matatini, New Zealand; 3 Landcare Research, Dunedin, New Zealand; 4 Departamento de Zoología, Campus de Rabanales, Universidad de Córdoba, Córdoba, Spain; 5 Epilab, Hopkirk Research Institute, Massey University, Palmerston North, New Zealand

**Keywords:** home-range, network model, *χ*^2^ distribution

## Abstract

While heterogeneity in social behaviour has been described in many human contexts it is often assumed to be less common in the animal kingdom even though scale-free networks are observed. This homogeneity raises the question of whether the patterns of behaviour necessary to account for scale-free social contact networks, where the degree distribution follows a power law, i.e. a few individuals are very highly connected but most have only a few connections, occur in animals, or whether other mechanisms are needed to produce realistic contact network architectures. We develop a space-utilization model for individual animal behaviour to predict the individuals' social contact network. Using basic properties of the *χ*^2^ distribution we present a simple analytical result that allows the model to give a range of predictions with minimal computational effort. The model results are tested on data collected in New Zealand for the social contact networks of the wild brushtail possum (*Trichosurus vulpecula*). Our model provides a better prediction of network architecture than other simple models, including a scale-free model.

## 1. Introduction

The social behaviour of animals can have a dramatic effect on the architecture of their contact networks. For example, controlling the transmission of disease on scale-free or clustered networks requires different strategies than when control is applied to a more homogeneous network [1]. It has long been recognized that the simple and analytically attractive random Erdős–Rényi (ER) network [2] is a poor description of many real-world, in particular social contact, networks [3]. Modern theory has popularized the scale-free (SF) network with its power-law degree distributions, which has given rise to many useful and accurate predictions about social networks [4]. However, the premise for a scale-free network is that individuals behave in remarkably different ways, with some exhibiting highly sociable behaviour with a vast number of friends or contacts, while others prefer a more solitary lifestyle. While such heterogeneity has been described in many human contexts, including disease transmission [5], behaviour is often assumed to be more homogeneous in the animal kingdom [6], even though scale-free networks are still observed [7]. This raises the question of whether the patterns of behaviour necessary to account for scale-free social contact networks occur in animals, or whether we need other mechanisms to produce realistic contact network architectures.

When a social network has been observed it is not ‘the’ network, but rather an example of a large number of possible networks that could have been observed depending on the exact observational circumstances [8]. To this end it is useful to develop models that can create suites of networks that all share similar properties. Statistical techniques can then be used to determine if an observed network could plausibly have been derived from that particular class of model. An ER network can be derived from the premise that any two individuals on the network have an equal chance of being in contact [2]. One mechanism that can be used to explain the patterns seen in a SF network is that when a new individual joins the network they are more likely to contact individuals who already have a large number of contacts, i.e. the rich get richer and the poor get poorer [4].

Here, we use social-contact data collected as part of a research project investigating disease transmission among wild free-living brushtail possums (*Trichosurus vulpecula*) [9] and a model based on home-ranges to create example contact networks. The brushtail possum has the widest native distribution of any Australasian marsupial, and is one of the most adaptable species in its native range [10]. Outside of its native range, in New Zealand, it is a major pest impacting native biodiversity [11], and is the primary wildlife reservoir of bovine tuberculosis (TB) [12]. Possums in New Zealand occur in densities ranging from 0.4 to 12 per hectare, depending on the habitat type [13], with reasonably well-defined home-ranges of a size negatively correlated with density [14]. For example, home-range area is commonly between 0.5 and 2 hectares in high density populations in native forest, but can reach up to 100 ha in generally low-density populations in more open pasture areas [15], with variation also driven by other factors including sex and age [16]. Wild possums are traditionally considered largely solitary animals [17]; however, with no social groupings, they often have widely overlapping home-ranges (both between and within sexes), in which interactions among individuals occur [18].

In our model of home-range areas, we assume possums have well-defined home-ranges centred at a random point in space. Individuals move within their home-range following an uncorrelated random walk and by chance come into contact with other individuals. We use this space-utilization model to define a contact network between individuals within a given area. We test the efficacy of our model by comparing the architecture of the home-range model with homogeneous (ER) and scale-free models and also a swap randomization scheme [19]. Our model is not without precedent. For example, Ramsey & Efford [20] used a similar but far more complex model to study the spread of TB through a possum population. Formica *et al*. [21] used a similar technique based on home-range data to investigate social-contact networks of forked fungus beetles, but their study again was fundamentally numerical and used a more complex methodology. Our approach differs significantly, in that we employ a simplified model in which a range of powerful analytic techniques can be employed to give estimates of biological parameters over wide ranges. In contrast to generating numerical simulations for each case of interest, this analytical approach allows exploration of a wider range of network properties and paves the way for more in-depth exploration of network dynamics and their underpinning mechanisms in future analyses.

## 2. Data collection

The study population inhabits a 1200 ha research area in the Orongorongo Valley (lower North Island, New Zealand (lat. −41°21′; long. 174°58′)). The site, made up of mixed native broadleaf-conifer forest, is a long-term study site for the management of TB in possums [22], and supports moderately high possum densities (approx. 7 ha^−1^) [16]. Each of four study sites (A, B, C and D) consisted of a *ca* 13 ha trapping grid, made up of 100 traps at 40 m spacing. The traps used were Grieve wire cage traps (60 × 26 × 28 cm) with spring-assisted folding doors triggered by a pendulum bait hook [23]. Traps were set on the ground, and baited each morning with apple sprinkled with powdered sugar and flour lured with anise oil. Monthly trapping sessions were conducted every four weeks during the study period (April–November 2012), each session consisting of traps being opened and checked for four consecutive nights at each site (with sprung traps reset, and possums recaptured during a trapping session only identified and released). When first captured, possums were anaesthetized by intra-muscular injection of Zoletil 100^®^ (Virbac New Zealand Ltd, Auckland, New Zealand) [24], weighed to the nearest 25 g, sexed, ear-tagged with a numbered metal tag on each ear (National Band & Tag Co. size 3, Kentucky, USA), and released at the point of capture. During initial capture months, up to 40 adult possums (20 male and 20 female) on each trapping grid were fitted with a Sirtrack™ encounter proximity radio-collar that had combined VHF and UHF components and weighed 45 g. Juvenile possums were still growing, and thus not collared for animal ethics reasons. Collars were programmed to detect and record other collars within 1 m (collar identification code, and time and length of interaction), with a separation time of 1 s (i.e. an interaction ended if the collars were more than 1 m apart for more than 1 s).

For the first occasion of each monthly trapping session, each recaptured collared possum was anaesthetized as before, had collar information downloaded on site via cable connection to a notebook computer, and then released. Collars that did not function correctly were replaced. Data consisted of a separate file for each individual on a trapping grid, with each record in a file representing a contact between that individual and another individual at the same site. Each record contained the ID of the individual encountered, the time at which the encounter occurred, and the length of the encounter. In theory, every encounter should have been recorded twice (i.e. once in the file of each interacting individual); however, in practice only 60% of pairs had fully consistent records for both individuals. Inconsistent records are frequently generated by differing alignments of proximity collars to one another (unpublished data). When a contact was logged by a single collar in an interacting pair, the data from the single collar was used. When a contact was logged by both collars, but encounter lengths differed, an average value was used. All contact records from sites B and D were useable. At sites A and C, 34% and 3% of records respectively were discarded as either corrupt or because the encounter length was longer than 1000 min. Self-contacts (two individuals at site D) were also discarded.

## 3. Network construction

The period of time when a particular individual was collared we refer to as the *collar time*, and is defined as the period between the first and last recorded contacts with any other individual. Collar times varied, so some individuals had less time in which to record contacts than others. To account for this, we define the total *available time* for each pair of individuals in contact as the intersection of the collar times of the pair. The *contact time* for each pair of individuals is the sum of the durations of their encounters in the *available time*. Finally, we combine these last two measurements to give the dimensionless variable, *relative contact time T_ij_*, the fraction of the total available time during which two individuals are in contact:

$$T_{ij}=\frac{\text{contact}\;\text{time}}{\text{available}\;\text{time}},$$

where *T_ij_* is the proportion of time individual *i* was in contact with individual *j*, and *T* is the matrix defining the contact network. (Note that *T* is a symmetric matrix in which *T_ii_* = 0 for all *i* as self-contacts have been discarded). This definition gives four separate contact networks for this study, one for each site. Note that these are contact networks with edges weighted by the relative amount of time two individuals are in contact.

**Figure 1.**
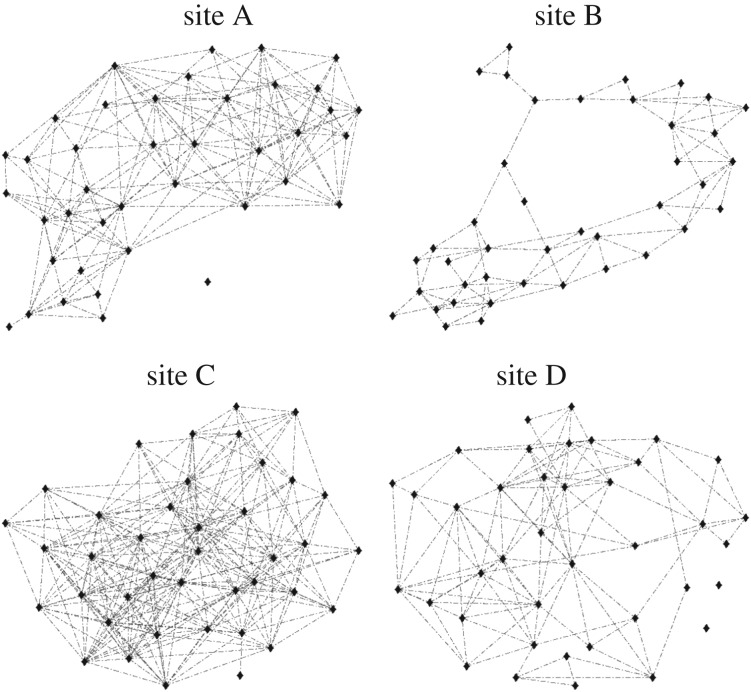
A visual representation of the social network at each site. Each point is an individual, lines between individuals show that those two individuals had a contact during the observational period. Note that the position of each individual does not represent their actual location at the site.

The four empirical networks, the contact networks of the possums at the four sites, are shown in figure 1, and several summary statistics are given in table 1. At each site there were approximately 40 individuals. At two sites (B and C) there were no individuals with zero network connections (i.e. all individuals were in contact with at least one other individual), while there were one and two unconnected individuals at sites A and D respectively. The expected proportion of individuals to which another individual is connected is commonly referred to as the *connectance* of the network. Note that in graph theory this is more usually termed the graph density but, in an ecological context, density is more often used to describe the number of individuals in a given area. We therefore use *connectance* to avoid confusion. The *degree* of an individual is the number of individuals to which it is connected. Site B has the lowest *connectance*; on average an individual has *degree* 4.2, giving a *connectance* of 0.11. Site C has the highest *connectance*, 0.37, and the average *degree* of an individual is 14.4.

**Table 1.** Summary statistics for the architecture of the empirical network at each of the four field sites. Estimates of home-range radius and area are based on Poisson distributed individuals within a square region, and a threshold *contact time* of 5 × 10^−8^.

site	network size	*connectance*	mean *degree*	unconnected individuals	length-3-cycles	estimated home-range radius (m)	estimated home-range area (ha)
A	40	0.232	9.1	1	296	59.5	1.1
B	41	0.105	4.2	0	47	36.0	0.4
C	40	0.368	14.4	0	884	81.7	2.1
D	39	0.161	6.1	2	126	46.9	0.7

## 4. Spatial model

We assume that each individual follows a mean reversion random walk. This results in each individual occupying a circular territory or home-range and moving only within this home-range. As the individual strays toward the edge of the home-range, the probability of the next movement being directed towards the centre of the home-range is increased. This is an Ornstein–Uhlenbeck process and can be described by the stochastic differential equation

$$\text{d}X_{t}=-\alpha (X_{t}-X_{0})\;\text{d}t+\beta \;\text{d}W_{t}.$$

The parameter *α* governs the strength of the ‘pull’ the individual feels back towards the centre of its home-range, and *β* governs the role of noise in the system (i.e. an individual with large *β* will move with a more random appearance). When applied to a two-dimensional walk with centre (*x*_0_, *y*_0_), the stationary (i.e. long-term) solution for this equation is the two-dimensional Gaussian probability distribution:

$$f(x,y)=\frac{\alpha }{\pi \beta^{2}}\exp\left(-\frac{\alpha ({\left(x-x_{0}\right)}^{2}+{\left(y-y_{0}\right)}^{2})}{\beta^{2}}\right).$$

This distribution has mean *μ* = (*x*_0_, *y*_0_) and variance *σ*^2^ = *β*^2^/2*α*. We define the home-range radius, *H*, of an individual to be *H* = 4*σ*. Using this definition, an individual will be within a distance *H* of the centre of the home-range for approximately 95% of the time [25].

### 4.1. An individual-based approach

In the individual-based model, networks were created to resemble those in the data by positioning *N* individuals within a region of area *A*, giving a spatial density of *ρ* = *N*/*A*. The *i*th individual on the network has home-range radius *H_i_*, where *H_i_* is taken from a normal distribution *N*(*H*, 0.1*H*), i.e. home-ranges have mean *H* and coefficient of variation 10%.

A *contact* is defined as two individuals coming within distance *R* of each other. Using the positions of each pair of individuals and their home-ranges, a matrix of interaction times *T* can be found where *T_ij_* is the proportion of time that individuals *i* and *j* are in contact with each other, i.e. the *relative contact time* defined in §3 (Network construction). Sample networks and a distribution for *T_ij_* can be found by running repeated simulations where *N* individuals with randomly assigned home-ranges are placed within the region using a spatial Poisson process.

**Figure 2.**
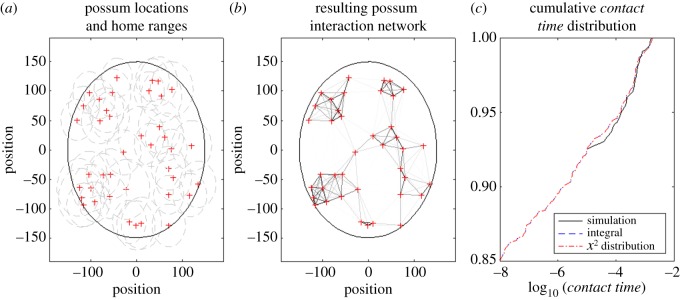
An example of the spatial model for possum contacts. (*a*) Each of 40 individuals (red crosses) are located within the region (black circle) and ascribed a home-range area (grey dashed circles). (*b*) The distance between neighbours determines the *contact time* (weight of network edge). (*c*) The cumulative distribution of *contact times* for all pairs of individuals on the network, as calculated by simulating the random walk for each individual (black line), using the integral of equation (4.2) (blue dashed line) and the *χ*^2^ method (red dot-dashed line).

Figure 2*a* shows the results from an example simulation with 40 individuals placed in a circular region with a 150 m radius. Each individual has an expected home-range radius of 40 m. The resulting network of interactions is shown in figure 2*b*, where the weighting of the network edges indicates the *relative contact time* between individuals. Figure 2*c* (solid black line) shows the cumulative frequency distribution of *contact times*. The simulation was run for 1000 time units with time steps of 0.01. Hence *relative contact times* below 10^−6^ are not possible.

### 4.2. Analytical results for *relative contact times*

As the position of each individual is defined by a normal distribution, the difference in their positions is also normally distributed. Without loss of generality we assume that individual *i* is centred at (0, 0) with home-range *H_i_* (i.e. variance $\sigma^{2}=H_{i}^{2}/16$) and individual *j* is centred at (*x*_0_, 0) with home-range *H_j_*. Therefore, the distance between them is normally distributed with mean (*x*_0_, 0) and variance $\sigma^{2}=(H_{i}^{2}+H_{j}^{2})/16$. The proportion of time they are in contact, *T_ij_*, is the probability that the distance between them is less than the contact distance *R*, and

4.1 $$\;T_{ij}=\iint_{C}\frac{1}{\pi \sigma_{2}^{2}}\exp\left(-\frac{\left({\left(x-x_{0}\right)}^{2}+y^{2}\right)}{\sigma_{2}^{2}}\right)\text{d}x\;\text{d}y,$$

where *C* is the circle of radius *R* centred at (0, 0). While this integral is not wholly analytically tractable, it can be simplified to a single integral and solved numerically using standard quadrature methods, albeit with strict tolerances required to give a desired level of accuracy. Figure 2*c* (blue dashed line) shows the cumulative distribution of *contact times* in the example simulation found using this integral.

A more useful analytical solution is to approach the problem using the non-central *χ*^2^ distribution. This distribution gives the sum of the squares of normally distributed random numbers with non-zero means and unit variance (i.e. for the sum of two numbers, this is a Euclidian distance squared). If we assume that both individuals have the same home-range, and rescale the problem so that the movement distributions have unit variance, we find that *T_ij_* is given by

4.2 $$T_{ij}\approx P\left(Y<\frac{R^{2}}{\sigma^{2}}\right)\;\text{where}\;\frac{Y^{2}}{\sigma^{2}}∼\chi^{2}\left(2,\frac{x_{0}}{\sigma^{2}}\right),$$

where *χ*^2^(*k*, *λ*) is the non-central *χ*^2^ distribution. This assumes that there is no correlation between the *x* and *y* position distributions of each individual, and in our spatial model individuals are moving in a ‘Brownian motion’ manner, so this lack of correlation is a valid assumption. Figure 2*c* (red dot-dashed line) shows the cumulative distribution of *contact times* in the example simulation found using this method. This analytical solution has the significant advantage of being highly amenable to numerical and analytic methods.

### 4.3. Quantifying the effect of sampling effort

Theoretically, under this model all pairs of individuals will be in contact with each other for a non-zero amount of time. However, pairs of individuals that are far apart have very low *relative contact times* and are very unlikely to be observed to meet in a finite observation period. To quantify this effect of sampling effort we assume that all values of $T_{ij}<\epsilon$ are zero. This assumption changes the model output from a fully connected network where every individual is predicted to meet every other individual—albeit with some very short *contact times* (i.e. very low edge weights)—to a partially connected network where some individuals are not connected.

In the case where each individual's position follows a spatial Poisson process (i.e. they are equally likely to be placed at any point in the region), the density of individuals does not affect the distribution of distances between pairs of individuals. Hence the distribution of interaction times is independent of population density, and theoretically only depends on the size of the region and each individual's home-range. In practice, if the home-ranges are small in comparison to the region, then only the home-range area has an impact on the distribution of interaction times. This is because interactions between pairs of individuals over large spatial distances become so small that they are discarded when the assumption that small interaction times ($T_{ij}<\epsilon$) are zero is applied.

**Figure 3.**
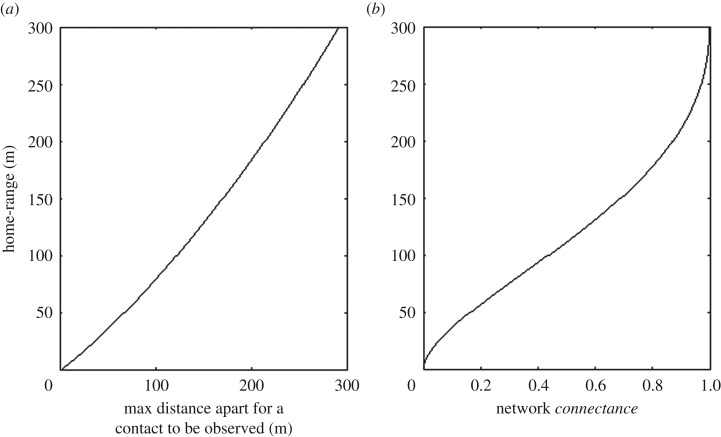
The spatial model can be used to predict the home-range radius of individuals on the network given the network *connectance*. Analytical results for the example network described in figure 1. (*a*) Given the home-range radius of two individuals, the maximum distance between their home-range centres such that they would be in contact for at least 10^−16^ of the observation period. (*b*) The relationship predicted by the spatial model between network *connectance*, i.e. the proportion of other individuals at the site (in this case a circle of radius 150 m) that an individual is likely to come into contact with, and the average radius of an individual's home-range.

Once a minimum *contact time* ϵ has been established, either theoretically or from data, we can use the *χ*^2^ method to calculate the maximum distance between the centres of two individual's home-ranges that would allow them to be observed to be in contact during the observational period for any given home-range area. Figure 3*a* shows this maximum distance for the scenario in figure 2, with a minimum *relative* *contact time* of 10^−6^ (i.e. as a proportion of the entire observation period) for a variety of home-ranges. As an example, the results in figure 2 predict that if we consider just those individuals with a home-range of 130 m who reside in a region of radius 300 m, then any given individual will be in contact with half of the other individuals. Furthermore, if individuals' home-ranges increase to 290 m, then all the individuals in the region will be in contact with each other. This calculation is not possible with the exact integral formulation as the tolerances required for the quadrature are numerically intolerable.

### 4.4. Predicting network *connectance*

If the distribution of distances between home-range centres is known, it can be used to predict the probability that any two individuals in the network have a *contact time* greater than the required minimum (i.e. the density of the network). For the example case in figure 2, we use the probability density function for the distance *x*_0_ between Poisson distributed points in a circle of radius *a* [26]

4.3 $$f(x_{0})=\frac{8x_{0}}{\pi a}\left(\cos^{-1}\frac{x_{0}}{2a}-\frac{x_{0}}{2a}{\left(1-{\left(\frac{x_{0}}{2a}\right)}^{2}\right)}^{1/2}\right).$$

For a given home-range, we then calculate the probability that a pair of individuals have home-range centres close enough to observe a contact (i.e. the network *connectance*). For the example in figure 2 this is shown in figure 3*b*, predicting that if individuals with a home-range of 300 m are placed randomly within a circle of 150 m radius, the resulting network will be fully connected (i.e. every individual will be in contact with every other). Note that this result is independent of the spatial density of individuals.

### 4.5. Model summary

We have presented a model that uses the location of individuals and information about their movement patterns to determine whether those individuals are in contact. Although the model is based on a stochastic process that is expensive to compute, we have also given a quick analytical solution that accurately predicts the proportion of time that two individuals will be in contact with each other. This can be used to generate example networks that represent the social interaction between individuals.

## 5. Results

To judge the efficacy of our spatial model, we use it to generate a number of outcomes that we compare with the empirical data.

### 5.1. Home-range prediction

Our first comparison is a simple one. As shown in figure 3*b*, assuming individuals with a given home-range are placed according to a Poisson process on a known region, the model predicts the *connectance* of the resulting network. Conversely, if the network *connectance* is known, we can predict the average home-range area of the individuals involved. As far as we are aware, this estimate of home-range area from network *connectance* is a novel one and is only made possible by using an analytical solution to the individual-based model such as the *χ*^2^ solution described here [27].

Our threshold *contact time*, $\epsilon =5\times 10^{-8}$, was chosen because this is twice the smallest *contact time* observed in the data. It corresponds approximately to one second in eight months (the study period). We assume that all individuals have the same home-range *H*. An equivalent figure to figure 3*b* was produced by calculating the minimum distance between home-range centres that would result in a *contact time* above the threshold for a range of home-range areas. The data observed individuals on a square region and there is no simple analytical equivalent of the Hammersley result, which uses a circular region. However, it is a simple matter to approximate this distribution numerically. Pairs of individuals (Poisson distributed) were chosen from a square region 300 × 300 m, and the proportion of pairs that were closer than the minimum distance needed for contact was calculated. This was conducted for a number of home-ranges and the predicted home-range for each study site was interpolated from these reference points. Table 1 shows the predicted home-range for each of the four sites. The predicted home-ranges have radii from 36 m to 82 m, equivalent to 0.4–2.1 ha.

### 5.2. Network architecture

To assess the power of the spatial model as a predictor of the architecture of the network (i.e. the structure but not the weight of the edges), it is necessary to have a null model for comparison. In network theory the choice of a null model is not always immediately obvious [28]. To this end we use three null models for comparison of the network architecture: a simple Erdős–Rényi (ER) randomization, a scale-free (SF) algorithm, and an algorithm that preserves degree distribution. All of the null models generate comparison networks that are the same size and, probabilistically, have the same *connectance*, as the empirical comparison network.

The ER null model generates a network where connections between individuals are assigned randomly, with all connections being as likely as all others. While the ER null model is pleasing in its simplicity, it is often criticized for ignoring any structure contained in the network. It has also long been discredited as a reasonable model for most real-world situations [3]. Our second null model is the scale-free model popularized by Barabasi *et al.* [29], as implemented by Newman [30]. This has received much attention since its inception and is considered a good model for social networks of many different types [4]. Our third null model produces networks with a given degree distribution and is based on the algorithm of Molloy & Reed [31]. This algorithm constructs a network by inserting connections randomly between individuals within the constraints of a given degree distribution. To allow fairer comparison between the model results and the data and randomizations, we carry out a separate comparison for each of the four empirical contact networks. For the spatial model we use the *connectance* of the data to estimate the home-range of individuals in the network as described above, and generate a suite of comparison networks with this *connectance*.

The model results (either spatial or the null models) were all taken from 1000 model realizations, and either by summarizing the results in the case of distributions or taking the expected result. To compare the accuracy of the spatial model in describing the architecture of the data, we calculated two different architecture metrics (degree distribution and number of cycles of length 3) and compared the predictions of the spatial model and the null models against the data. It is worth mentioning that in these comparisons simple *p*-value and confidence intervals are often meaningless. The comparison models are generated from stochastic realizations and, subject to time constraints, we can generate as many of these as we care to. As more realizations are generated, *p*-values and confidence intervals may become smaller and smaller. In preference to these measures, we use statistics that are robust to the number of realizations, such as the Kolmogorov–Smirnov or KS-statistic, inter-quartile range and standard deviation of distributions.

### 5.3. Degree distribution

Degree distribution is widely accepted as an important metric in real-world networks [32]. An Erdős–Rényi network gives a degree distribution that follows a Poisson distribution, while a network created using preferential attachment will have a degree distribution that follows a power law. We compare the degree distribution of the four empirical contact networks with the expected degree distribution from each of the comparison models. The given degree distribution randomization of Molloy & Reed [31] was excluded in this comparison, as it is designed to reproduce the degree distribution of the empirical contact networks. Each empirical contact network was compared separately with the model networks, and the expected degree distributions of each model were found by amalgamating the degree distributions from 100 model realizations.

**Figure 4.**
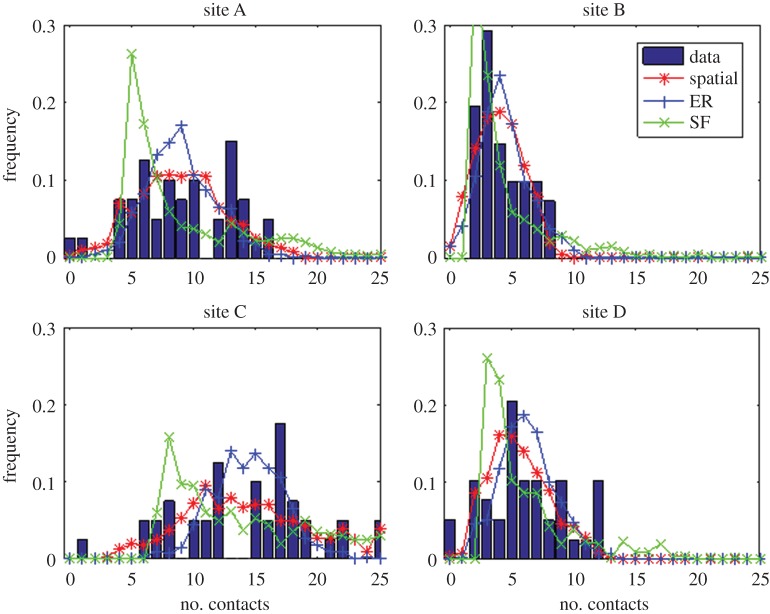
The spatial model provides a good prediction of the degree distribution of the possums' empirical interaction (or social) networks. The distribution of the number of contacts between individuals for each of the four empirical networks (blue bars). The degree distributions predicted by the spatial model (red stars) are the closest fit of the three candidates for all four cases. The Erdős–Rényi (blue +) and scale-free (green ×) models predict distributions that have a higher KS and RMSE statistic (table 2).

The models produced networks with the same *connectance* (probabilistically), and hence the same average degree, as the empirical contact network. Table 2 shows the results of the comparisons, and figure 4 shows the degree distributions. When the standard deviation of the model degree distributions is considered, none of the models conclusively stand out as a candidate for closest model. However, when the KS-statistic and root-mean-square error (RMSE) are used to measure the closeness of the model predicted degree distributions to the empirical data, the spatial model provides the best fit for all four study sites.

**Table 2.** Summary statistics of degree distribution for comparisons between the empirical networks and the model realizations. The closest standard deviation and smallest Kolmogorov–Smirnov statistic (KS) and root-mean-square error (RMSE) are highlighted for each empirical comparison. Italics denote the model with the closest fit as judged by that statistic. Judging by standard deviation, there is no conclusive ‘best’ model. However, both the KS statistic and RMSE suggest that the spatial model produces degree distributions that are closer to those of the empirical networks than either the scale-free or ER models.

		degree distribution			
site	model	mean	s.d.	KS comparison (empirical model) KS (*p*)	RMSE (empirical model)
A	data	9.1	4.1	—	—
	spatial		*3.6*	*0.11 (0.66)*	*0.035*
	ER		2.6	0.20 (0.07)	0.047
	SF		*4.6*	0.17 (0.17)	0.051
B	data	4.2	1.9	—	—
	spatial		*2.1*	*0.10 (0.79)*	*0.034*
	ER		*1.9*	0.11 (0.64)	0.040
	SF		3.2	0.18 (0.13)	0.048
C	data	14.4	5.6	—	—
	spatial		4.9	*0.10 (0.74)*	*0.036*
	ER		3.0	0.19 (0.1)	0.045
	SF		*5.7*	0.21 (0.05)	0.045
D	data	6.1	3.3	—	—
	spatial		2.5	*0.10 (0.81)*	*0.039*
	ER		2.3	0.11 (0.69)	0.041
	SF		*3.8*	0.22 (0.04)	0.071

### 5.4. Number of cycles

**Figure 5.**
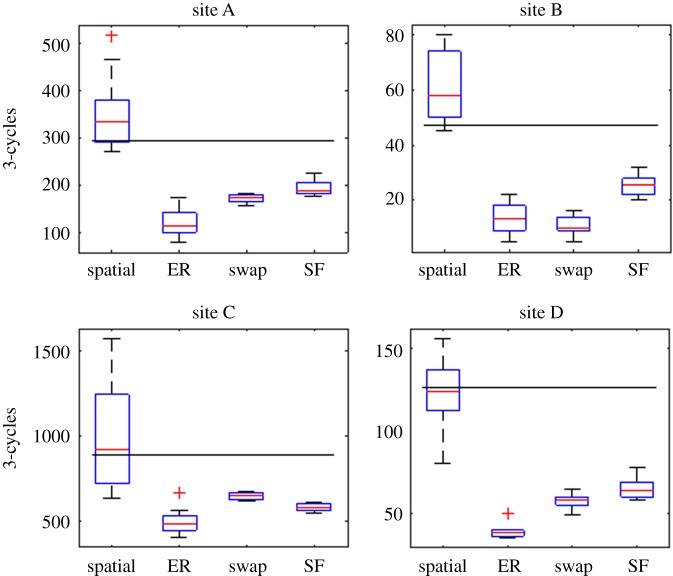
The number of 3-cycles seen in the empirical network data is best reproduced by the spatial model. Each box plot shows the distribution of cycles of length 3 seen in 100 realizations of each model. For three of the four sites, the number of 3-cycles seen in the empirical network falls within the interquartile range (box) of the spatial model prediction. The fourth site (B) is still within the 99% limit (whiskers) of the predicted distribution. Conversely, the ER, swap and SF models all significantly underpredict the number of 3-cycles observed.

It is well known that two networks can have the same degree distribution but still be markedly different [3,4]. To this end, other network metrics have been developed that can be used to quantify the properties of a network. A simple metric used in graph theory as an indicator of network structure is the number of cycles of length 3. A length-3-cycle occurs when individual A is connected to individual B, who is then connected to individual C, who in turn is connected to individual A. The number of length-3-cycles in each empirical network is shown in table 1. Figure 5 shows a box plot representing the distribution of the number of 3-cycles in each of the null models when compared with each of the four empirical networks. The box plot displays median, interquartile range, 99th percentile, and outliers. In three of four cases (sites A, C and D) the number of 3-cycles in the empirical contact networks is within the interquartile range of the spatial model distribution; for the fourth site (B) the data lie easily within the 99th percentile range. In contrast, the other three null models demonstrate markedly fewer 3-cycles than the empirical contact networks, and in no case does the number observed empirically fall inside the 99th percentile range. When the same analysis is conducted with the number of cycles of length 4, we see an almost identical pattern. This similarity in results for 3- and 4-cycles is not unexpected as the number of each of these is known to be strongly linked in most random graphs.

## 6. Discussion

We have presented a space-utilization model of animal behaviour that has been used to generate contact networks of individuals. These networks can be generated from either direct numerical simulation or from an analytical result based on the *χ*^2^ distribution. We have used *connectance*, a network characteristic, to estimate the size of the individual's home-range (i.e. 0.4–2.1 ha), which is realistic compared with the observed average home-ranges for possums at our study area of 1.3 ha and 1.8 ha for females and males respectively [16]. We have generated a suite of random networks and compared their characteristics to empirical data. The spatial model is a more plausible fit to the data than three other null models presented, including the popular scale-free model.

Scale-free networks have been widely and successfully used as models of social contact for a range of subjects [4]. They predict heavy-tailed degree distributions where the majority of individuals have only a few contacts but some individuals have many. They have underlying mechanisms that mimic real-world behaviour, e.g. preferential attachment, that add to their appeal as models. We have presented a real-world situation where the arising networks are not well described by a scale-free model. In fact they appear to be dominated by a scale that is fixed by the behaviour of an individual, i.e. its home-range. The space-utilization model presented here results in networks that are not scale-free as individuals with a very large number of contacts are unlikely to arise. Neither are they similar to the small-world networks put forth by Watts & Strogatz [33]. These networks are characterized by short path lengths between any two individuals on the network. It is clear that the space-utilization model will result in very long path lengths between individuals when the home-ranges are small and the spatial region is large. In particular it does allow the formation of ‘short-cuts’ between groups of individuals that are characteristic of small world networks.

A limitation of our individual-based model is the use of a spatial Poisson process to define the locations of individuals. On a large scale this process results in a homogeneous distribution of individuals but in each realization of the process there will be local areas of space with very few individuals and some areas with a higher local density (see for example figure 3*a*). In some respects this heterogeneity could be seen to be analogous of many landscapes where some areas offer better habitat for individuals than others. However, on the scale of the data collected here, it is more likely that individuals self-organize to use the space more uniformly. In this case we would expect the resulting degree distributions to have a lower variance than those predicted with the spatial Poisson process used here as all individuals become more similar. This would result in degree distributions which were even less similar to a scale-free distribution, which already has a higher variance than that predicted by the spatial model (table 2). However, network architecture is reliant on more than just degree distribution. Our results also show even when two networks have the same degree distribution they can still differ greatly even in basic metrics [34] such as the number of cycles measured here. Again, the spatial model gives a better prediction of the cycles metric than the other candidate network models. Variance between individuals will also be seen due to their spatial location at the field site, individuals at the edge will have fewer observed contacts than those at the centre. However the model does account for this as locations were spatially distributed within a square region.

We would expect intrinsic attributes of the species in question (e.g. sex, age, body weight, phylopatry behaviour, movements, etc.) to affect the social network [34] and consequently the architecture metric. Also females tend to settle near their maternal home-range resulting in adjacent females having a higher probability of being closely related. In the particular case of brushtail possums, we dealt with a solitary species whose contacts are primarily associated with mating (i.e. March and April, [23]) and less frequent at other times [35]. This work was part of a wider study [36] on how TB persists across the year, and thus was undertaken primarily outside the mating season. It is also known that there are differences in home-range area between males and females, juveniles and adults [16], and between populations inhabiting different habitats [13,15,16]. In particular juveniles have smaller home-ranges which are generally within the maternal home-range. For ethics reasons, juveniles could not be included in this study. The networks simulated here by the space-utilization model are limited to the more homogeneous case where all individuals have the same home-range. The analytical solution presented here, based on the non-central *χ*^2^ distribution, is strictly only valid when individuals have the same size home-ranges. However, numerical explorations show that the non-central *χ*^2^ distribution is still an excellent approximation when the home-ranges are allowed to vary by up to 10%. The results from the networks with this more varied behaviour are almost identical but computationally far more expensive as they must be generated from first principles rather than using the analytical results.

The spatial modelling of TB in possums used to underpin management actions in New Zealand has recently been made more realistic through the inclusion of metrics of home-range overlap [37], indicating that the network details elicited here do have real-world implications. Incorporating the network model we developed into management models is thus likely to further improve their accuracy. An obvious next step for this work is to use the *χ*^2^ distribution to predict the amount of time that two individuals spend in contact. However, we expect this to be less successful as the amount of time two individuals spend together is most likely dictated by the behaviour of individuals. For example when two animals meet they may fight, mate or simply run away, and these behavioural choices are less successfully modelled with a naive random walk. It will be interesting to see if once this aspect of behaviour is considered, the resulting networks may have some scale-free characteristics, particularly in the edge weights rather than the edge architecture.

## Data accessibility

Data are available through the Landcare Research Data depository at https://doi.org/10.7931/J24Q7S5N.
